# Foreign Correspondence

**Published:** 1846-07

**Authors:** 


### THE WESTERN JOURNAL.

New Series.—Vol. VI.—No. I.

LOUISVILLE, JULY 1, 1846.

### FOREIGN CORRESPONDENCE.

*London*, May 16th, 1846.

Gentlemen:—

I have supposed that an account of the curriculum of study, prescribed by the *University of London* for degrees in medicine, would be interesting to the readers of the Western Journal. I am the more inclined to adopt this mode of introducing myself to them from the fact, that my stay in London has been too short to allow of my going into details respecting any of the various hospitals in which this vast metropolis abounds; and I am not yet sufficiently acquainted with the *magnates* in our profession who have congregated in London, to jot down any new or instructive facts in relation to their history, practice, or merits. What I write is the substance of the *Regulations of the University of London for Degrees in Medicine.*

The student, entering the University, produces a certificate to the effect that he has completed his sixteenth year. He is then examined on the following subjects:—Mathematics, natural philosophy, chemistry, the classics, the English language, and outlines of history and geography. He shall not be approved by the Examiners unless he show a competent knowledge of the classics, including the English language, history and geography, mathematics, and either natural philosophy or chemistry. This is the matriculation examination, as it is called.

The examination for the degree of Bachelor of Medicine, requires that the candidate shall have been engaged during four years in his professional studies at one or more of the institutions recognised by the University. He is also obliged to certify that he has completed his nineteenth year; that he has taken a degree in Arts in this or some recognised University, or has passed the matriculation examination; that he has been a student two years at one or more of the medical institutions acknowledged by this University, subsequently to having taken a degree in arts, or passed the matriculation examination; that he has attended a course of lectures on four of the subjects embraced in the following list:—Descriptive and surgical anatomy, general anatomy and physiology, comparative anatomy, pathological anatomy, chemistry, botany, materia medica and pharmacy, general pathology, general therapeutics, forensic medicine, hygiene, midwifery and diseases peculiar to women and children, surgery, and medicine. In addition to this it is necessary that he shall have dissected nine months; have attended a course of practical chemistry, and have given sufficient attention to practical pharmacy to enable him to prepare medicines. The fee for this examination is $25; that for the former is $10.

The subjects upon which the candidates are examined are anatomy, physiology, chemistry, botany, materia medica, and pharmacy. It is in this part of the course that "exhibitions" and gold medals are awarded to the successful candidates for honors.

The candidate who most distinguishes himself, and who evinces sufficient merit in anatomy and physiology, or in chemistry, or materia medica and pharmaceutical chemistry, receives an "exhibition" of $150 per annum for the next two years. Under the same circumstances the first and second candidates in each branch receive a gold medal of the value of $25. After studying two years longer, the student is allowed to become a candidate for the second examination for M.B., after he has produced certificates to the following effect: of having passed the first examination, and subsequently to this, attended a course of lectures on at least two of the subjects embraced in the list above mentioned, and for which he had not presented certificates at the first examination; of having, after the first examination dissected six months; of having attended the surgical practice of a recognised hospital during other twelve months, and lectures on clinical surgery; of having attended the medical practice of a recognised hospital during other twelve months, and lectures on clinical medicine; of having, subsequently to these two years attendance upon surgical and medical practice, attended to practical medicine in a hospital or dispensary, during six months. The candidate is examined upon physiology, general pathology, general therapeutics and hygiene, surgery, medicine, midwifery, and forensic medicine. The fee for this, as for the first examination for M.B., is $25. Here, as in the United States, all fees are returned if the candidate be unsuccessful.

Scholarships, valued at $250 per annum, for two years, and gold medals estimated at $25, are given to the successful candidates for honors.

The examination for the degree of Doctor of Medicine, which is conferred in the United States upon candidates who have studied twenty-four months, is thus conducted here:—No candidate is admitted to this examination unless he have produced certificates which go to show that he has taken the degree of M.B. in this, or a degree in medicine or in surgery, at some other respectable University; of having attended, subsequently to having taken one of the degrees in medicine, to clinical or practical medicine during two years in a hospital; or, of having attended one year in a hospital, and been engaged for three years in the practice of his profession. Or if he have taken the degree of M.B. in this University, he shall have been engaged during five years in the practice of his profession.

The candidate is examined upon elements of intellectual philosophy, logic, moral philosophy, and medicine. He is allowed to choose the subject of his Thesis. A gold medal worth $50 is awarded to the writer of the best dissertation.

Examinations for honors are allowed upon surgery, medicine, and midwifery. A gold medal valued at $25 is the prize of the successful competitor.

From what I have said you will learn one, and the principal one, of the causes which give Englishmen such rank in the profession of medicine.

The time necessary to become physicians is at shortest more than twice as long as that required in the United States. In order to pass the matriculation, the candidate is obliged to be acquainted with mathematics, natural philosophy or chemistry, the classics, including the English language, history, and geography. In order to attain the degree of Bachelor of Medicine, he submits himself to two other examinations in order to be a candidate, for the first of which he shall have studied four years, nine months of which time he must have dissected. To be eligible for the second examination he is to have attended lectures and hospital practice for two years subsequently to his having undergone the first, to have conducted six labors, and dissected six months.

Six years have been already devoted to his studies, and he has only arrived at the second step, that of M.B. If he would go regularly on and become a Doctor of Medicine, he must continue his attendance upon hospital practice for two years longer. The entire period, from the time that he passes his matriculation examination until he is an M.D., is eight years.

It is sometimes, I am told, performed in less time in the instance of young men who have taken several prizes, and exhibited marked sedulousness in their studies. From the published Regulations of the University College of London, I see no provision of this kind; though, I suppose, it is not to be doubted that there are instances in which the entire eight years' study is not required. Some of the students who are now in attendance at the college express themselves to me as anticipating an apprenticeship of eight years; others, again, are confident of becoming physicians at an earlier period.

Among the eminent medical men of London I have already had the pleasure of meeting Mr. Lawrence and Mr. Liston, the first of whom is surgeon to Bartholomew's and the other to the University Hospital. Mr. Lawrence is an elderly gentleman, of fine person, large head, and expansive forehead, and in blandness and courteousness of manners is a good specimen of the "fine old English gentleman." He visits the hospital twice a week, and I shall make a point of being always present at his clinics. You rarely see so much size and physical strength combined with such rapidity of movement in the same person as you are struck with in Mr. Liston. As he moves about over the city he drives ahead like one putting out fire; and his walk is characteristic of the man, rapid and untiring. When in his carriage his horses go in a sweeping trot; when he alights from it he runs rather than walks; in the wards of the hospital he glances at a patient and seems to perceive by intuition the nature of his disease and the remedy for it; he asks but few questions, makes a few remarks, enjoins simple and brief directions, and then moves on to another. His business at the hospital dispatched, he runs back to his carriage and whirls away to attend to his large business. But, with all this celerity of motion, he does nothing in a hurry; but whatever he undertakes he performs thoroughly and well. His organization is eminently a happy one. If he were a slothful man, he would soon grow fat, and becoming so, would cease to exert himself sufficiently to maintain that preëminent station which he now justly occupies among the surgeons of this great metropolis. He is a Scotchman by birth, nearly six feet high, of a herculean frame, and still in the prime of philosophical life. He has performed the operation of lithotomy one hundred and forty times, and that of lithotripsy sixty times. I shall have more to say of this great surgeon in my next.

